# Association between Sleep Duration and Overweight/Obesity at Age 7–18 in Shenyang, China in 2010 and 2014

**DOI:** 10.3390/ijerph15050854

**Published:** 2018-04-25

**Authors:** Qi Sun, Yinglong Bai, Lingling Zhai, Wei Wei, Lihong Jia

**Affiliations:** Department of Child and Adolescent Health, School of Public Health, China Medical University, Shenyang 110122, China; sunqi@cmu.edu.cn (Q.S.); ylbai@cmu.edu.cn (Y.B.); llzhai@cmu.edu.cn (L.Z.); wwei@cmu.edu.cn (W.W.)

**Keywords:** children, adolescent, sleep duration, overweight, obesity

## Abstract

This study was designed to examine the association between sleep duration and being overweight/obese in primary, middle, and high school students. This was a multiple cross-sectional study using data from the 2010 and 2014 National Survey on Students’ Constitution and Health (CNSSCH). A total of 23,602 students aged 7–18 years were enrolled in this study. The prevalence of being overweight and obese—stratified by age, gender, and sleep duration—in 2010 and 2014 were compared. Sleep duration was categorized as <7 h, ≥7 to 8 h, ≥8 to 9 h, and ≥9 h. Overweight and obesity were defined according to the cut-point criteria in China. Multivariable logistic regression results in 2010 and 2014 revealed that students sleeping <7 h and aged 7–12 years had an increased risk of becoming overweight/obese. In 2010, the adjusted prevalence ratios of overweight for 7–12-year-old students sleeping <9 h was 1.196 (95%CI: 1.004–1.424) and 13–15-year-old students sleeping <8 h was 1.265 (95%CI: 1.023–1.565). In 2014, the adjusted prevalence ratios of overweight and obesity for 7–12-year-old students sleeping <9 h were 1.295 (95%CI: 1.091–1.537) and 1.231 (95%CI: 1.045–1.449); 16–18-year-old students sleeping <7 h were 1.530 (95%CI: 1.239–1.888) and 1.585 (95%CI: 1.270–2.081). Our study revealed that different levels of sleep curtailment increased the risk of becoming overweight/obesity in different age groups of students.

## 1. Introduction

The prevalence of being overweight and obesity among children and adolescents has been rising at a staggering rate during the last 30 years [1,2]. There were 41 million overweight children in the world in 2014, about 10 million more than there were two decades ago [3]. The results of a 2015 report on Chinese nutrition and chronic disease showed that, in 2002, the proportions of children aged 6–17 years who were obese and overweight in China were 2.1% and 4.5%, respectively. Those numbers increased to 6.4% and 9.6% in 2012. Childhood obesity often follows into adulthood and links to many serious consequences, including impaired glucose tolerance, hypertension, and cardiovascular diseases. Furthermore, childhood obesity can cause important psychological impacts such as academically poor performance, social stigma, laziness, and depression and anxiety [4,5,6].

Specifically, obesity is a complex condition which can be influenced by a wide range of factors. Besides the genetic factors, diet, energy expenditure, and television viewing are known to contribute to an increased risk of being overweight and obesity [7,8,9]. Recently, shorter sleep duration in infancy and childhood has been regarded as a risk. It has been proposed that sleeping problems are causally relative to obesity in early life [10,11,12]. The associations between sleep duration and obesity in different age groups have been reported in some cross-sectional and cohort studies [13,14,15]. A prospective cohort study reported that children aged 2.5–6 years old who consistently slept less than 10 h were at 2.9 times the risk of becoming obese as compared to those who consistently slept 11 h or more [16]. Another cohort study assessed the association between sleep duration in childhood and adult body mass index (BMI) in a birth cohort, finding that shorter sleep duration was significantly associated with higher adult BMI values [17]. However, there is limited information on the relationship between sleep duration and obesity among children and adolescents in China, which especially lacks valuable data derived from extremely cold areas with high prevalence of obesity. 

Taken together, we used the data obtained from National Survey on Students’ Constitution and Health (CNSSCH) sampling in Shenyang to investigate whether sleep duration is a risk factor of being overweight or obesity in children and adolescents. An apparent strength is that there has not been any large-scale study reporting an association between sleep duration and obesity/overweight in Shenyang with winter lasting more than 6 months (the coldest temperature is approximately −30 °C).

## 2. Materials and Methods

### 2.1. Study Participants

The data were obtained from and approved by CNSSCH, which conducted two samplings in 2010 and 2014 in Shenyang, a northeastern city in China. The survey performed by CNSSCH is complex, multistage, cross-sectional, and nationwide. There has been a standardized methodology, which has become a continuous survey, since 1985, and data have been released every 4 or 5 years. The subjects in the present study were primary and secondary school students aged 7–18 years, randomly selected from urban and rural residential areas in Shenyang according to the requirements of CNSSCH sampling. The physical examinations and sampling methods were the same as the protocols used for previous surveys in all CNSSCH studies [18,19]. Therefore, the data derived from the 2010 and 2014 CNSSCH surveys in Shenyang were comparable and reliable depending on two indicators: (1) all the participants were uniformly measured by using the same methods and in the same way for the two years and (2) all the participants were school students aged from 7 to 18 years who were enrolled by stratified cluster random sampling from Shenyang, according to the Handbook of National Student Physical Health Survey in 2010, excluding those students diagnosed with abnormal growth and development or physical abnormalities. The participants were stratified by gender (male or female, with approximately equal numbers in each gender) and evenly distributed into different socioeconomic classes (upper, middle, and lower). The sample size of each survey was 11,528 and 12,074 in 2010 and 2014, respectively.

In this analysis, children and adolescents were classified into three age groups (7–12, 13–15, and 16–18 years), the age ranges commonly adopted to differentiate students for primary, middle, and high school in Shenyang, China, respectively.

### 2.2. Anthropometric Measurements

Height and weight examinations of students were performed in schools according to the protocol criteria [20]. All measurements at the survey site were conducted by trained professionals.

The measuring instruments of height (cm) and weight (kg) at all survey sites were similar [20]. Height was measured to an accuracy of 1 mm using a calibrated stadiometer (TZCS-4, Co., Ltd. Xinman Science and Education Equipment, Shanghai, China) and weight was measured to an accuracy of 0.1 kg using a calibrated leveraged scale (RGT-140, Co., Ltd. Xinman Science and Education Equipment). Students were required to wear light clothing without shoes and stand erectly when measuring height. The height and weight of each student were measured twice, and the average numbers were recorded to decrease error. BMI was calculated as the ratio of weight (kg) to height squared (m^2^), which is considered as an effective indicator to assess adiposity. Obesity and overweight were defined according to the Working Group of Obesity in China [21]. 

### 2.3. Questionnaire Survey

The questionnaire, which has been carried out since 2010, was designed by the CNSSCH group and mainly surveyed the lifestyle habits influencing growth and health of children in order to put forward prevention measures for promoting the growth of children and adolescents. As an important lifestyle, daily sleep duration was reported by students or parents voluntarily in the questionnaire, and the relationship between sleep duration and overweight/obesity was explored in this study. Inspectors who were familiar with the questionnaire went to schools and distributed the questionnaire. The completed questionnaires were checked for quality control. For primary school students, one of the parents would answer the questionnaire on the student’s behalf to prevent inaccurate information. Questionnaires were distributed in class. The team ensured that students would bring back the answered questionnaire to school. When all of the questionnaires were handed in, researchers collected them from each class, and quality control was performed. Sleep duration was categorized as <7 h, ≥7 to 8, ≥8 to 9 h, and ≥9 h. 

Based on the data reported by Shen and his colleagues that the average sleep duration of primary school students was 9 h and 10 min, 8 h and 6 min for middle school students, and 7 h and 9 min for high school students [22], in the present study, sleep curtailment was defined as sleep duration less than 9 h for 7–12 year age group; less than 8 h for 13–15 year age group; and less than 7 h for 16–18 year age group.

### 2.4. Statistical Analyses

The percentages of obesity and being overweight among different gender and age groups in 2010 and 2014 were described and a χ^2^ test was adopted for categorical variables. As a remarkably different prevalence and incidence of overweight/obesity between boys and girls was observed, stratified analysis based on gender was performed to examine the sleep–overweight/obesity association according to different genders. Furthermore, sleep duration changes as a child grows to adulthood and generally decreases when children get older, therefore, stratified analysis based on different age groups was also performed to examine the sleep–overweight/obesity association according to different age group.

Odd ratios (ORs) were used to assess the cross-sectional association between sleep duration and risk of overweight/obesity. Multivariate logistic regression was performed to select potential covariates for the dependent variable (obesity or overweight). Normal-weight children were selected as a reference for the dependent variable. The independent variables (age group, gender, and sleep duration) were entered into logistic regression separately; the adjustment OR for one variable was counted and the other two variables were adjusted. Except where otherwise specified, a two-tailed *p* value < 0.05 was considered significant. Data were analyzed by Statistical Package for the Social Sciences (SPSS, version 20.0, IBM Corporation, New York, NY, USA).

### 2.5. Declaration

Ethics approval was granted by the China Medical University, and the study was conducted in accordance with the ethics standards of the Committee on Human Experimentation. The consent procedure was approved by the Ethics Committee of the China Medical University.

## 3. Results

### 3.1. Demographic Characteristics

Demographic distributions were presented in Table 1. A total of 23,602 children and adolescents were included in the final analysis, which were recruited from primary, middle and high schools in Shenyang, China. In 2010, 50.43% and 49.83% of the students were boys for 2010 (5814) and 2014 (5714), respectively, whereas 49.57% and 50.17% were girls for 2010 (6016) and 2014 (6058), respectively. There were no significant differences in the distribution of number by gender and age group in 2010 and 2014.

### 3.2. Prevalence of Obesity and Overweight in 2010 and 2014

As shown in Table 2, the prevalence of obesity and overweight was 8.59% and 15.28% in 2010, respectively, and 13.18% and 15.61% in 2014, respectively. In 2010, the detection rate of obesity in the 7–12-year-old group was significantly higher than the other age groups, moreover, that in 16–18-year-old group was significantly lower than 13–15-year age group (*p* < 0.001). The variation trend of obesity rate in 2014 was similar to 2010. Moreover, the detection rates in each age group in 2014 were significantly higher than in 2010 (*p* < 0.001), whereas there was no difference among age groups in 2010 and 2014.

Data in Table 3 showed that the prevalence of obesity and overweight in boys was significantly higher than girls both in 2010 and 2014 (*p* < 0.001), the obesity rates of boys and girls increased significantly in 2014 as compared to 2010 (*p* < 0.001).

### 3.3. Association between Sleep Duration and Prevalence of Obesity and Overweight

Table 4 shows sleep duration stratified by age group for 2010 and 2014, respectively. The results revealed that the prevalence of obesity was different among sleep duration groups in 2010 and 2014 (*p* < 0.001). Moreover, there was a significant difference in sleep duration among different age groups in both 2010 and 2014 (*p* < 0.001; shown in Table 5). 

### 3.4. Multivariate Logistic Regression Model Predicting Obesity and Overweight

Based on the results mentioned above, age, gender and sleep duration might be having an influence on overweight and obesity. Therefore, they were enrolled as independent variables for multivariate logistic regression, in which obesity and overweight were dependent variables and normal weight was selected as a reference. The results presented in Table 6 indicates that the multivariable adjusted ORs (95%CI) for overweight in 2010 were 1.294 (95%CI: 1.077–1.555) and 1.259 (95%CI: 1.057–1.500) in sleep duration for <7 h and ≥7 to 8 h, respectively, in comparison with ≥9 h; 1.683 (95%CI: 1.516–1.868) in boys in comparison with girls; 1.425 (95%CI: 1.225–1.658) and 1.176 (95%CI: 1.025–1.349) in the 7–12 and 13–15 age groups, respectively, in comparison with the 16–18 age group. However, there was no significant trend for the association between sleep duration and obesity. The multivariable-adjusted ORs (95%CI) for obesity were 1.916 (95%CI: 1.671–2.196) in boys in comparison with girls; 2.823 (95%CI: 2.291–3.478) and 1.671 (95%CI: 1.365–2.045) in the 7–12 and 13–15 age groups, respectively, in comparison with the 16–18 age group. 

In 2014, the multivariable adjusted ORs (95%CI) for overweight were 1.405 (95%CI: 1.177–1.677), 1.265 (95%CI: 1.060–1.509), and 1.253 (95%CI: 1.050–1.495) for sleep duration <7 h, ≥7 to 8 h, and ≥8 to 9 h, respectively, in comparison with ≥9 h; 1.883 (95%CI: 1.700–2.085) in boys in comparison with girls; 1.474 (95%CI: 1.278–1.699) and 1.148 (95%CI: 1.002–1.316) in 7–12 and 13–15 age groups, respectively, in comparison with the 16–18 age group. The multivariable adjusted ORs (95%CI) for obesity were 1.308 (95%CI: 1.093–1.564) for sleep duration <7 h in comparison with ≥9 h; 1.953 (95%CI: 1.749–2.182) in boys in comparison with girls; 2.714 (95%CI: 2.306–3.195) and 1.483 (95%CI: 1.258–1.749) in 7–12 and 13–15 age groups, respectively, in comparison with the 16–18 age group.

### 3.5. Effect of Sleep Curtailment on Obesity and Overweight Stratified by Age Group

The results presented in Table 7 reveal that sleep curtailment (sleep duration <9 h) was positively associated with overweight in the 7–12-year age group, with multivariable adjusted OR 1.196 (95%CI: 1.004–1.424), but not related (*p* = 0.515) to obesity in 2010. For 2014, the adjusted ORs in sleep curtailment in the 7–12-year age group were 1.295 (95%CI: 1.091–1.537) and 1.231 (95%CI: 1.045–1.449) for overweight and obesity, respectively. Results for the 13–15-year age group (shown in Table 8) were similar to the 7–12-year age group, with the adjusted ORs of overweight in sleep curtailment (sleep duration <8 h) was 1.265 (95%CI: 1.023–1.565) in 2010, whereas there was no significant relationship (*p* = 0.912 and 0.504 for overweight and obesity, respectively) between sleep curtailment and obesity in 2014. Data in Table 9 indicate that sleep curtailment (sleep duration <7 h) was positively associated with overweight and obesity in the 16–18 year age group, with multivariable adjusted ORs 1.530 (95%CI: 1.239–1.888) and 1.585 (95%CI: 1.270–2.081), respectively, in 2014, but no obvious relevance reflected in 2010 (*p* = 0.870 and 0.340 for overweight and obesity, respectively).

## 4. Discussion

The present study provided valuable insight into the effect of sleep duration on prevalence of obesity and overweight among school-aged children and adolescents in Shenyang, located in northeast China. Firstly, our study indicated that the obesity rate of students in 2014 was significantly higher than in 2010, the average increase rate was 1.15% during 4 years, much higher than the national average [23]. The prevalence of obesity and overweight was different among age groups and gender. Obesity rate in the 7–12-year age group was much higher than in other age groups, moreover, the obesity and overweight rates for boys was 4.36% and 5.48%, respectively, higher than for girls in 2010, while the values in 2014 were nearly twice that of girls. 

Secondly, similar to previous reports suggesting that short sleep durations could be an independent risk factor for obesity, our results indicated that the prevalence of obesity was different among <7 h, ≥7 to 8, ≥8 to 9 h, and ≥9 h sleep duration groups [24,25,26], and sleep duration of students obviously reduced as their ages advanced. In our study, more than half of the students in all three age groups did not sleep more than 9 h and nearly 60% of high school students slept less than 7 h, far less than the recommended sleep duration (9–11 h) for children and adolescents [27]. Therefore, we hypothesized that overweight/obesity might be affected by sleep duration, thus multivariate logistical regression was performed to analyze association between sleep duration and overweight/obesity. Using sleep duration of more than 9 h as a reference, data in 2010 revealed that participants with sleep duration <7 h and ≥7 to 8 h were, respectively, 1.294 and 1.259 times more likely to be overweight. In 2014, participants with sleep duration <7 h, ≥7 to 8 h, and ≥8 to 9 h were, respectively, 1.405, 1.265, and 1.253 times more likely to be overweight, and participants with sleep duration <7 h were 1.308 times more likely to have obesity. In addition, data in both 2010 and 2014 indicated that the younger the age, the greater the risk of obesity and overweight. In the two multivariate logistical regressions, the contribution of age was the most for obesity and the contribution of gender was the most for overweight. Moreover, the schoolwork burden of primary, middle, and high school students in China is increasing, and one consequence is that sleep duration of each age group has been reduced (2010 vs. 2014). Therefore, age group might be a confounding factor for the association between sleep duration and overweight/obesity. 

In the third part of our study, multivariate logistical regressions were performed, adjusting for different age groups, to explore the association between sleep curtailment and obesity/overweight. For the 7–12-year age group, sleep duration of less than 9 h was adjudged as sleep curtailment, which increased the risk of becoming overweight/obese in 2010 and 2014. For the 13–15-year age group, sleep curtailment (<8 h) only added the risk of becoming overweight in 2010. For the 16–18-year age group, sleep duration of <7 h might increase the risk of becoming overweight/obese by 1.530/1.585 times, respectively, in 2014. Our findings that curtail of sleep was associated with higher risk of being overweight and obesity in children and adolescents is consistent with previous cross-sectional studies [15,28]. Furthermore, the additional new insight from the present study was that it classified different divisions of sleep deprivation for primary, middle, and high school students, which will be more suitable for actual situations of education in primary and secondary schools in China. 

There are several speculations that have been proposed to elaborate on the mechanisms causing sleep curtailment to increase the risk of overweight/obesity. A previous study has demonstrated that sleep restriction may reduce the excretion of growth hormone (GH) [29]. For children, GH is secreted during sleep and promotes body height and inhibits the lipoprotein lipase activity of adipose tissue, which can reduce the risk of overweight/obesity [30]. The effects of decreased GH secretion induced by sleep curtailment are more likely to be related to the influence on lipoprotein lipase activity than length growth [31]. Furthermore, reduced leptin and elevated ghrelin were observed in a large sample of adults, which was associated with increased hunger and appetite—especially for high carbohydrate content, including sweets, salty snacks, and starchy foods—which in turn could alter the balance of energy intake and energy expenditure [32,33,34]. Hjorth et al. [35] also demonstrated that a 1 h decrease in sleep duration increased the intake of added sugar and sugar-sweetened beverages. Another explanation may be presumed that children with a shortened sleep duration may have fewer physical activities because of daytime tiredness, whereas corresponding more sedentary time could affect the night sleep quality [36,37]. In addition, it should be considered that obesity-related sleep apnea could result in sudden awakening at night and reduce sleep duration; however, sleep apnea could also be an independent risk factor of obesity [38]. However, the present cross-sectional study could not address the causal relationship between sleep apnea and obesity. 

Studies in adults have explored the U-shaped association between long or short sleep duration and obesity [39,40]. However, few studies have discovered a similar U-shape for children and adolescents. Our study did not explore the effect of prolonged sleep duration on childhood and adolescent overweight/obesity, as we mainly focused on the risk of obesity induced by sleep curtailment in primary, middle, and high school students. Furthermore, there are various causes of sleep curtailment in school-aged children and adolescents including TV watching and computer use, studying burden, family numbers, socioeconomic differences, and so on. These will be explored in our further study [41,42]. 

## 5. Conclusions

This study first defined sleep curtailment—adjusting for primary, middle, and high school students—as less than 7, 8, or 9 h, respectively. Different levels of short sleep duration for 7–12-, 13–15-, and 16–18-year age groups increased the risk of becoming overweight/obese. Optimizing sleep duration for different levels of students may be an important intervention for becoming overweight and obese. Further research will explore the association between sleep duration and leptin/ghrelin in children and adolescents to deeply dig out underlying mechanisms of obesity.

## Figures and Tables

**Table 1:** Characteristics of study population in 2010 and 2014.

	Age (Year)			Total
	7–12	13–15	16–18	
2010				
Boys	2296	1793	1725	5814
Girls	2165	1783	1766	5714
Total	4461	3576	3491	11,528
2014				
Boys	2562	1807	1647	6016
Girls	2564	1766	1728	6058
Total	5126	3573	3375	12,074

**Table 2:** The prevalence of obesity and overweight among age groups in 2010 and 2014 (%).

	Age Group (Year)	Normal	Overweight	Obesity	Total	χ^2^	*p*
2010	7–12	3206 (71.87)	720 (16.14)	535 (11.99)	4461	140.95	0.000
	13–15	2747 (76.82)	549 (15.35)	280 (7.83) *	3576		
	16–18	2823 (80.87)	493 (14.12)	175 (5.01) *#	3491		
	Total	8776 (76.13)	1762 (15.28)	990 (8.59)	11,528		
2014	7–12	3385 (66.04)	838 (16.35)	903 (17.62) &	5126	186.89	0.000
	13–15	2612 (73.10)	554 (15.51)	407 (11.39) *&	3573		
	16–18	2601 (77.07)	493 (14.61)	281 (8.33) *#&	3375		
	Total	8598 (71.21)	1885 (15.61)	1591 (13.18)	12,074		

Note: * vs. 7–12 years, *p* < 0.001; # vs. 13–15 years, *p* < 0.001; & vs. 2010, *p* < 0.001.

**Table 3:** The prevalence of obesity and overweight among gender in 2010 and 2014 (%).

	Gender	Normal	Overweight	Obesity	Total	χ^2^	*p*
2010	Boys	4132 (71.07)	1057 (18.18)	625 (10.75)	5814	167.62	0.000
	Girls	4644 (81.27)	705 (12.34) ^	365 (6.39) ^	5714		
	Total	8776 (76.13)	1762 (15.28)	990 (8.59)	11,528		
2014	Boys	3890 (64.66)	1147 (19.07)	979 (16.27) &	6016	251.08	0.000
	Girls	4708 (66.49)	738 (10.42) ^	612 (8.64) ^&	7081		
	Total	8598 (71.21)	1885 (15.61)	1591 (13.18)	12,074		

Note: ^ vs. boys, *p* < 0.001; & vs. 2010, *p* < 0.001.

**Table 4:** The prevalence of obesity and overweight among different sleep duration groups in 2010 and 2014 (%).

	Sleep Duration	Normal	Overweight	Obesity	Total	χ^2^	*p*
2010	<7 h	2956 (77.85)	579 (15.25)	262 (6.90)	3797	37.37	0.000
	≥7–8 h	2485 (76.34)	510 (15.67)	260 (7.99)	3255		
	≥8–9 h	1908 (74.01)	403 (15.63)	267 (10.36)	2578		
	≥9 h	1427 (75.18)	270 (14.23)	201 (10.59)	1898		
	Total	8776 (76.13)	1762 (15.28)	990 (8.59)	11,528		
2014	<7 h	3238 (72.10)	718 (15.99)	535 (11.91)	4491	35.89	0.000
	≥7–8 h	2419 (72.40)	525 (15.71)	397 (11.88)	3341		
	≥8–9 h	1646 (68.27)	387 (16.05)	378 (15.68)	2411		
	≥9 h	1295 (70.73)	255 (13.93)	281 (15.35)	1831		
	Total	8598 (71.21)	1885 (15.61)	1591 (13.18)	12,074		

**Table 5:** Different sleep duration among age groups in 2010 and 2014 (%).

	Age Group (Year)	<7 h	≥7–8 h	≥8–9 h	≥9 h	Total	χ^2^	*p*
2010	7–12	416 (9.32)	880 (19.68)	1617 (36.39)	1548 (34.61)	4461	4000.09	0.000
	13–15	1243 (34.24)	1346 (37.83)	721 (20.17)	266 (7.75)	3576		
	16–18	2138 (61.08)	1029 (29.55)	240 (7.01)	84 (2.36)	3491		
	Total	3797 (32.94)	3255 (28.24)	2578 (22.36)	1898 (16.46)	11,528		
2014	7–12	848 (16.54)	1070 (20.87)	1636 (31.92)	1572 (30.67)	5126	3613.78	0.000
	13–15	1489 (41.67)	1283 (35.91)	606 (16.96)	195 (5.46)	3573		
	16–18	2154 (63.82)	988 (29.27)	169 (5.01)	64 (1.90)	3375		
	Total	4491 (37.20)	3341 (27.67)	2411 (19.97)	1831 (15.16)	12,074		

**Table 6:** Summary of multivariate logistic regression model predicting obesity and overweight in 2010 vs. 2014.

		Variables	β	Wald	Sig.	OR ^a^ (95% CI)
2010	Overweight	Sleep duration (h)				
		<7 h	0.258	7.559	0.006	1.294 (1.077–1.555)
		≥7 to 8 h	0.230	6.638	0.010	1.259 (1.057–1.500)
		≥8 to 9 h	0.161	3.385	0.066	1.174 (0.990–1.393)
		≥9 h (reference)	0 ^b^			
		Gender				
		Boys	0.521	95.809	0.000	1.683 (1.516–1.868)
		Girls (reference)	0 ^b^			
		Age group (Year)				
		7–12	0.354	21.065	0.000	1.425 (1.225–1.658)
		13–15	0.162	5.351	0.021	1.176 (1.025–1.349)
		16–18	0 ^b^			
	Obesity	Sleep duration (h)				
		<7 h	0.145	1.586	0.208	1.157 (0.922–1.450)
		≥7 to 8 h	0.088	0.674	0.411	1.092 (0.885–1.348)
		≥8 to 9 h	0.109	1.165	0.280	1.115 (0.915–1.360)
		≥9 h (reference)	0 ^b^			
		Gender				
		Boys	0.650	87.112	0.000	1.916 (1.671–2.196)
		Girls (reference)	0 ^b^			
		Age group (Year)				
		7–12	1.038	94.820	0.000	2.823 (2.291–3.478)
		13–15	0.513	24.812	0.000	1.671 (1.365–2.045)
		16–18	0 ^b^			
2014	Overweight	Sleep duration (h)				
		<7 h	0.340	14.142	0.000	1.405 (1.177–1.677)
		≥7 to 8 h	0.235	6.826	0.009	1.265 (1.060–1.509)
		≥8 to 9 h	0.225	6.268	0.012	1.253 (1.050–1.495)
		≥9 h (Reference)	0 ^b^			
		Gender				
		Boys	0.633	147.862	0.000	1.883 (1.700–2.085)
		Girls (Reference)	0 ^b^			
		Age group (Year)				
		7–12	0.388	28.503	0.000	1.474 (1.278–1.699)
		13–15	0.138	3.934	0.047	1.148 (1.002–1.316)
		16–18	0 ^b^			
	Obesity	Sleep duration (h)				
		<7 h	0.268	8.632	0.003	1.308 (1.093–1.564)
		≥7 to 8 h	0.078	0.734	0.391	1.081 (0.904–1.293)
		≥8 to 9 h	0.164	3.425	0.064	1.178 (0.990–1.401)
		≥9 h (Reference)	0 ^b^			
		Gender				
		Boys	0.670	140.860	0.000	1.953 (1.749–2.182)
		Girls (Reference)	0 ^b^			
		Age group (Year)				
		7–12	0.999	144.096	0.000	2.714 (2.306–3.195)
		13–15	0.394	21.951	0.000	1.483 (1.258–1.749)
		16–18	0 ^b^			

^a^ Adjustment odds ratio (OR); ^b^ as a reference.

**Table 7:** Association between sleep curtailment and overweight or obesity stratified by age group in 2010 and 2014 (7–12-year age group).

		Variables	β	Wald	Sig.	OR ^a^ (95%CI)
2010	Overweight	Sleep curtailment				
		Yes	0.179	4.012	0.045	1.196 (1.004–1.424)
		No	0 ^b^			
		Gender				
		Boys	0.770	80.074	0.000	2.159 (1.824–2.556)
		Girls (Reference)	0 ^b^			
	Obesity	Sleep curtailment				
		Yes	0.064	0.424	0.515	1.067 (0.878–1.295)
		No (Reference)	0 ^b^			
		Gender				
		Boys	0.619	41.707	0.000	1.857 (1.539–2.240)
		Girls (Reference)	0 ^b^			
2014	Overweight	Sleep curtailment				
		Yes	0.258	8.737	0.003	1.295 (1.091–1.537)
		No (Reference)	0 ^b^			
		Gender				
		Boys	0.826	106.086	0.000	2.284 (1.952–2.672)
		Girls (Reference)	0 ^b^			
	Obesity	Sleep curtailment				
		Yes	0.208	6.193	0.013	1.231 (1.045–1.449)
		No (Reference)	0 ^b^			
		Gender				
		Boys	0.561	54.555	0.000	1.752 (1.510–2.033)
		Girls (Reference)	0 ^b^			

^a^ Adjustment OR; ^b^ as a reference.

**Table 8:** Association between sleep curtailment and overweight or obesity stratified by age group in 2010 and 2014 (13–15-year age group).

		Variables	β	Wald	Sig.	OR ^a^ (95%CI)
2010	Overweight	Sleep curtailment				
		Yes	0.235	4.727	0.030	1.265 (1.023–1.565)
		No (Reference)	0 ^b^			
		Gender				
		Boys	0.271	8.322	0.004	1.312 (1.091–1.578)
		Girls (Reference)	0 ^b^			
	Obesity	Sleep curtailment				
		Yes	0.278	3.578	0.059	1.320 (0.990–1.761)
		No (Reference)	0 ^b^			
		Gender				
		Boys	0.508	15.657	0.000	1.661 (1.292–2.136)
		Girls (Reference)	0 ^b^			
2014	Overweight	Sleep curtailment				
		Yes	0.012	0.012	0.912	1.013 (0.812–1.262)
		No (Reference)	0 ^b^			
		Gender				
		Boys	0.466	24.148	0.000	1.593 (1.323–1.918)
		Girls (Reference)	0 ^b^			
	Obesity	Sleep curtailment				
		Yes	0.087	0.446	0.504	1.091 (0.845–1.408)
		No (Reference)	0 ^b^			
		Gender				
		Boys	0.663	36.297	0.000	1.940 (1.564–2.407)
		Girls (Reference)	0 ^b^			

^a^ Adjustment OR; ^b^ as a reference.

**Table 9:** Association between sleep curtailment and overweight or obesity stratified by age group in 2010 and 2014 (16–18-year age group).

		Variables	β	Wald	Sig.	OR ^a^ (95%CI)
2010	Overweight	Sleep curtailment				
		Yes	−0.016	0.027	0.870	0.984 (0.808–1.197)
		No (Reference)	0 ^b^			
		Gender				
		Boys	0.461	21.840	0.000	1.586 (1.307–1.925)
		Girls (Reference)	0 ^b^			
	Obesity	Sleep curtailment				
		Yes	0.156	0.912	0.340	1.169 (0.848–1.611)
		No (Reference)	0 ^b^			
		Gender				
		Boys	1.056	37.888	0.000	2.875 (2.054–4.024)
		Girls (Reference)	0 ^b^			
2014	Overweight	Sleep curtailment				
		Yes	0.425	15.663	0.000	1.530 (1.239–1.888)
		No (Reference)	0 ^b^			
		Gender				
		Boys	0.506	25.834	0.000	1.658 (1.364–2.015)
		Girls (Reference)	0 ^b^			
	Obesity	Sleep curtailment				
		Yes	0.460	10.972	0.001	1.585 (1.270–2.081)
		No (Reference)	0 ^b^			
		Gender				
		Boys	1.052	59.840	0.000	2.864 (2.194–3.738)
		Girls (Reference)	0 ^b^			

^a^ Adjustment OR; ^b^ as a reference.

## Abbreviation

CNSSCH: National Survey on Students’ Constitution and Health
